# Colloidal Silver in Bronchopneumonia

**Published:** 1907-09

**Authors:** 


### Colloidal Silver in Bronchopneumonia.

G. Bjorkman, A.M., M.D., Professor of Physiology, Milwaukee Medical College, says: (*An Index of Diseases*), regarding the treatment of bronchopneumonia: the bacterial trio—the strepto-, staphylo- and pneumococcus—is the main cause of bronchopneumonia. Fortunately we have a remedy with very active offensive properties to all three, especially to the streptococcus, which is the sole instigator of the severest type of bronchopneumonia,—colloidal silver in concentrated or half-concentrated solution given rectally or intravenously. The speedy descent of the fever curve is remarkable. When two or three ounces of the solution (one-third for children) is given morning and night, with hydrotherapy, bronchopneumonia sometimes loses its foothold at once and yields with a willingness comparable only to diphtheria under antitoxin treatment. This treatment should, therefore, always be resorted to, even when a case seems hopeless. The remedy should be given sufficintly long to guard against relapse and sequelæ. If there are complications, the silver treatment may not always be successful; but in uncomplicated forms, especially when the streptococcus is in the lead and the remedy is applied early, it is almost a specific. The prognostic views of bronchopneumonia—hitherto so gloomy—will be considerably modified if collargolum gains more popularity in the disease.

Under "Lobar Pneumonia" Prof. Björkman states: the same holds good in every respect with lobar pneumonia. Even if a direct action on the pneumococcus in certain cases is less conspicuous, the visible mitigation of mixed infection is important enough to make the remedy an indispensable adjuvant.

Prof. Björkman also warmly recommends collargol in polyarthritis, as also in the various forms of sepsis, such as in infectious tonsilitis, lymphangitis, phlegmonous processes, pemphigus neonatorum, scarlatina, diphtheria, and the like. It should be given intravenously in severe erysipelas, meningitis cerebrospinalis, perimetritis, and appendicitis.

